# Temperature-Induced Internal Stress Influence on Specimens in Indentation Tests

**DOI:** 10.3390/mi13071045

**Published:** 2022-06-30

**Authors:** Shunbo Wang, Xianke Li, Hongwei Zhao

**Affiliations:** 1 School of Mechanical and Aerospace Engineering, Jilin University, Changchun 130025, China; wangshunbo@jlu.edu.cn (S.W.); lixk21@mails.jlu.edu.cn (X.L.); 2 Key Laboratory of CNC Equipment Reliability, Ministry of Education, Changchun 130025, China; 3 Mechanical Engineering College, Beihua University, Jilin 132000, China

**Keywords:** indentation, temperature environment, test error, internal stress, FEA

## Abstract

The factors affecting the internal stress of specimens during indentation tests were investigated by finite element analysis (FEA) modelling. This was carried out to gain a qualitative understanding of the test errors introduced by the temperature environment during the indentation process. In this study, the influence of thermal expansion of fixed stage on upper specimen (currently neglected in temperature indentation) was explored in detail. Technical issues associated with the parameters of the specimen (such as thickness, width, and elastic modulus) and external conditions (such as stage and glue) were identified and addressed. The test error of the calculated hardness and elastic modulus of the specimen reached up to more than 3% simultaneously at −196 °C (temperature of liquid nitrogen). Based on these considerations, the preferred operation conditions were identified for testing in specific temperature environment. These results can guide experiments aimed at obtaining precise mechanical parameters.

## 1. Introduction

Indentation technique has been extensively used in material science [1], microelectronics engineering [2], and condensed matter physics [3] owing to its high accuracy, low requirements for samples, and unique stress loading distribution. With the expansion of application fields, especially in aerospace engineering, conventional indentation devices have been utilized in diverse temperature environment, forming elevated temperature and low temperature indentation techniques. The testing temperature can reach 1600–10 K [4,5]. Numerous phenomena, distinguished from room temperature (RT), have been discovered [6,7]. The mechanical parameters obtained through temperature indentation are employed to guide the reliable development of engineering fields from the micro point of view. Thus, the application of indentation tests performed at various temperatures has become increasingly important.

Not only the properties of material are related with the way of loading [8] and external environment [9], but the result of indentation test is also influenced easily by the depth of indentation (size effect) [10], the way of loading [11] and internal environment. Thus, to ensure the change of test result is the change of property of material at different temperature, we should also consider the different of the accuracy of indentation load-displacement curves (*P*-*h* curves) at various temperature for comparison with that at RT. It is important that the uniformity of temperatures between indenter and specimen be guaranteed at both elevated and low temperature indentation tests [12,13]. The temperature difference will inevitably cause contact thermal drifting during the penetration process, as well as inaccurate and unreliable *P*-*h* curves [14]. Meanwhile, the testing environment including oxygen-free [15] and steam-free [16] environment (usually implemented using vacuum chamber), is also considered in indentation to ensure that the specimen surface maintains its initial properties. However, most investigations deem test results are accurate, provided that the experiments can be conducted favorably, and the *P*-*h* curves seem “normal”. In reality, for test results to be accurate, some potential but important influential factors should also be considered. For example, the anisotropy of the indenter-induced thermal expansion variation of area function and elastic modulus of the indenter at different temperatures. This can affect the calculations of the hardness and elastic modulus of a material [17], resulting in inherent errors up to more than 3%.

In this study, we employed a finite element analysis (FEA) of indentation to understand how the internal stress of specimens on a deformed fixed stage (under varying temperatures) influences the results of indentation tests in current experiments. The internal stress in the indentation region depends on the difference between the thermal expansion coefficients of the specimen and the downward fixed stage. It is difficult for specific experimental tests to exert post correction processes, as the additional stress depends on the parameters of specimens, fixed stage, adopted glue, and temperature simultaneously. Individual change in one parameter can result in transformation from tensile stress to compressive stress. Significant changes were observed in the *P*-*h* curves and were used to calculate mechanical parameters (hardness and elastic modulus) of specimen considering internal stresses at different temperatures.

## 2. Finite Element Model

**Figure 1:**
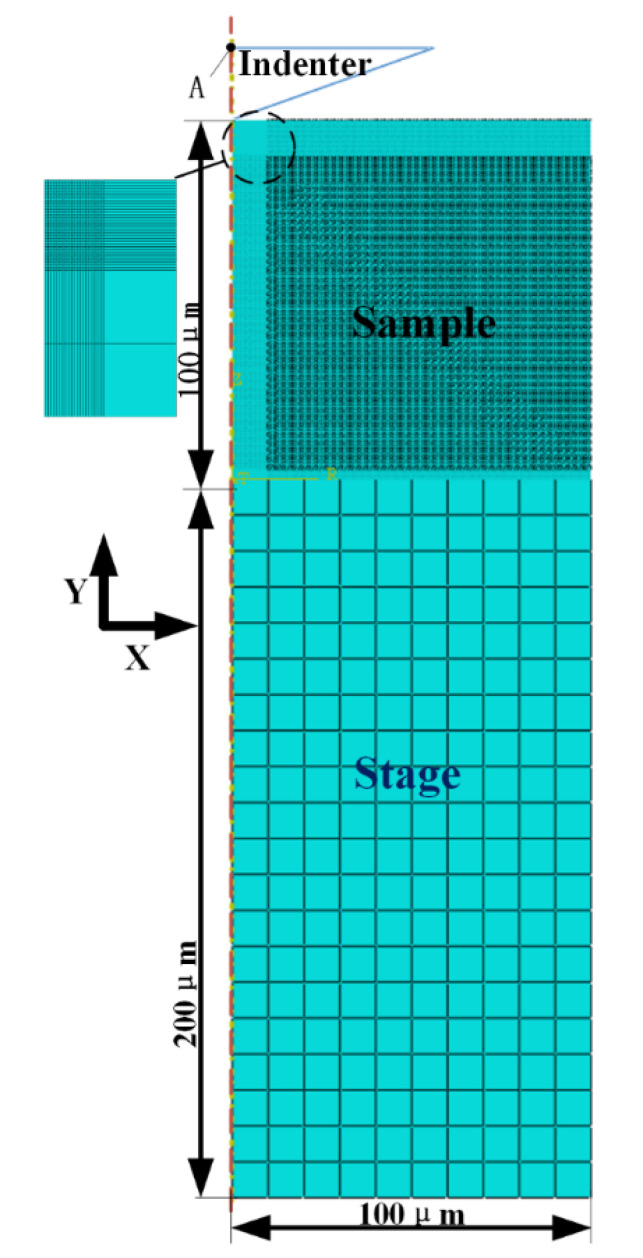
Representative finite element model in the current work.

Commercial simulation software ABAQUS (Dassault Systemes, Paris, France) was used for modelling and analysis for this study. The overall model is shown in Figure 1. All parts in the figure are meshed with an axisymmetric 4-node bilinear element CAX4T. Lichinchi et al. [18] demonstrated that there is a slight difference between the results obtained from two-dimensional (2D) axisymmetric and three-dimensional (3D) finite element simulations of nanoindentation experiments. Thus, the modelling method significantly reduced simulation time while ensuring accurate results. The half-angle of the adopted indenter was 70.3°. This had the same projected area-depth function as that of the standard Bosch indenter [19]. To reduce the influence of the indenter’s own properties on this experiment, the indenter was defined as an analytical rigid body. Point A was set as a reference point. The size of the sample was 100 × 100 μm (thickness × width). It was divided into 133,600 cells. For accuracy and efficiency considerations, the area closer to the indentation region (where penetrated occurred) was represented by denser grid. In other regions, the grid was appropriately enlarged. The size of the stage was 200 × 100 μm (thickness × width). The specific dimensions of the sample and stage could be adjusted in corresponding simulations. However, since it was not the focus of study, the grid setting was slightly larger and was divided into 200 cells in total. The *X*-direction displacement fixed constraint was applied to the symmetry axis of all parts, while the *Y*-direction displacement and *Z*-direction rotation fixed constraints were applied to the bottom of the stage. To simulate the ideal adhesive scenario, a binding constraint was imposed at the bottom of the sample and top surface of the stage. Note that the indenter was in hard contact with the top surface of the sample without friction. the contact surface of indenter was also used as the main surface. Unless otherwise specified, the material settings were the same as the overall model. An aluminum alloy (AlCu_2.5_Mg) was set as the material of the sample and manganese steel (A333–1.6) was the material of the stage. The specific properties of the materials are listed in Table 1.

A four-step simulation process was followed in this study. Initially, the temperature of the sample and the stage were both 20 °C and the indenter was separated from the sample. With displacement control, the temperature–displacement indentation process was simulated in four steps. Step 1: the sample and the stage simultaneously expanded to a stable temperature; Step 2: the indenter was pre-contacted with the sample; Step 3: the indenter was pressed 800 nm into the sample; and Step 4: the indenter was elevated to its original position. Point A in Figure 1 is used to extract the load and displacement value during indentation.

## 3. Results

**Figure 2:**
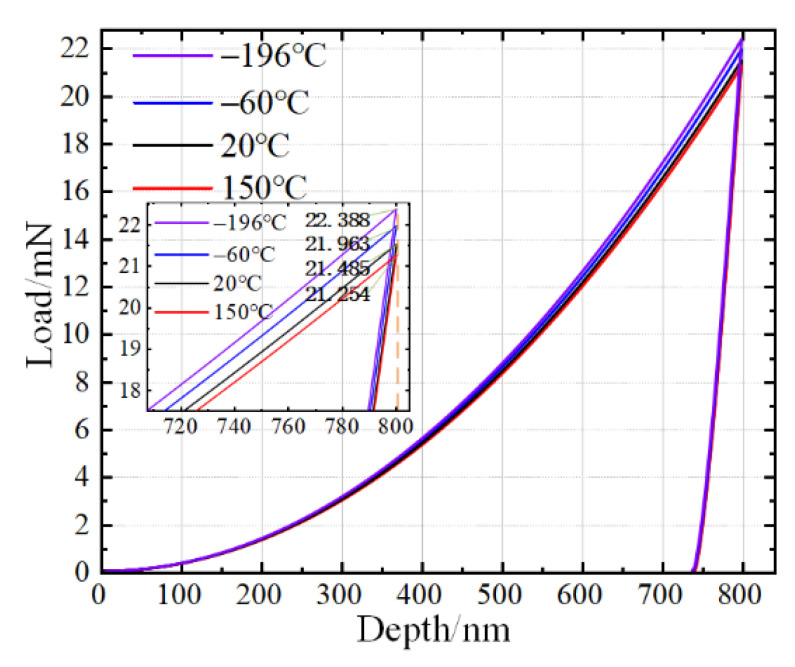
Indentation curves considering internal stress assuming that mechanical properties of material remain unchanged with temperatures.

Variable temperature indentation tests were conducted at −196, −60, 20, and 150 °C with the maximum indentation displacement of 800 nm using the model shown in Figure 1. The material properties are listed in Table 1. The displacement and force during the indentation process were extracted using the indenter reference point A and the indentation curves were drawn as shown in Figure 2. It should be noted that the adopted mechanical properties of the AlCu_2.5_Mg sample were the same at all temperatures. This was unachievable in experimental research. Under this setting, the indentation curves (a representation of the mechanical properties) should be coincident with each other. However, it was seen that the maximum loads at −196, −60, 20, and 150 °C were 22.388, 21.963, 21.485, and 21.254 mN, respectively. Moreover, the corresponding material hardness and elastic modulus changed in the different cases. According to the Oliver–Pharr theory, the calculated mechanical parameters of the specimen and corresponding error percentage are shown in Table 2. It was evident that the error percentage of hardness and elastic modulus reached up to 4.32 and 3.27% at −196 °C, respectively, which indicated that any mechanical properties obtained from experiments conducted at liquid nitrogen temperature had an inherent error of more than 3%. This has been overlooked in earlier studies and is unacceptable in indentation tests.

**Figure 3:**
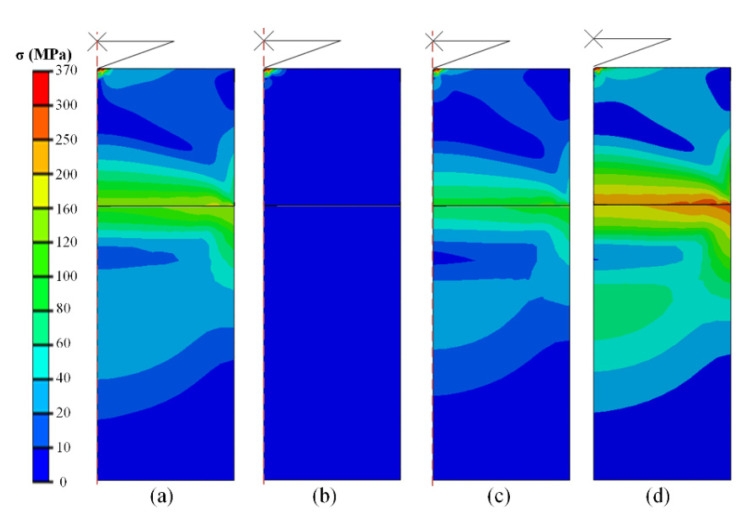
Stress distribution inside the specimen and stage during indentation at: (**a**) 150 °C; (**b**) 20 °C; (**c**) −60 °C; and (**d**) −196 °C.

Figure 3 shows the distribution of the Von Mises stress inside the specimen and downward fixed stage during the indentation process at different temperatures. It is evident that there is no other stress on the specimen and stage at 20 °C except the region affected by the indentation process. This was an ideal and normal test condition. However, significant internal stress occurred inside the two objects with temperature variations. This made the maximum stress distribute around the interface between the specimen and stage indicating that the difference of thermal expansion coefficient between the sample and stage induced interactive forces (further, transfers to the surface of the sample at the indentation region). In addition, the value of the test temperature had significant influence on the internal stress values. The large temperature difference from 20 to −196 °C (Figure 3b,d) induces more intense stress distribution, while the elevated and low temperatures led to a difference of positive and negative values of error percentages for the calculated mechanical properties, as listed in Table 2. More influential factors and specific influence law, including parameters of the specimen and external conditions, will be discussed in detail in the next section.

## 4. Discussion

### 4.1. Specimen Parameters

**Figure 4:**
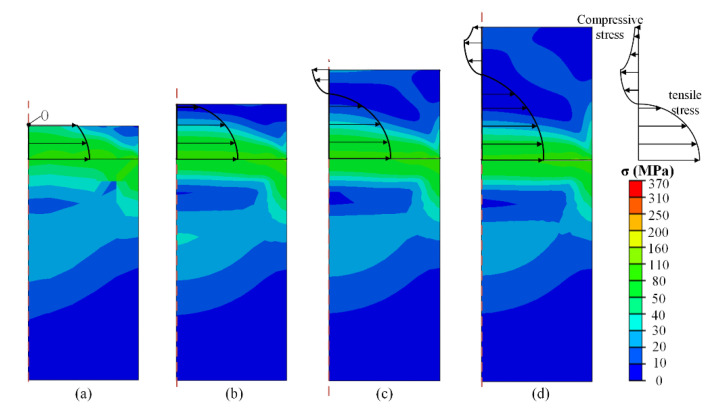
Stress distribution in specimen with thickness of specimen of (**a**) 0.3 mm; (**b**) 0.5 mm; (**c**) 0.8 mm; (**d**) 1.2 mm at −60 °C at the condition of 72 GPa elastic modulus of specimen.

To explore the distribution law of stress with the shape parameter of the specimen, the thickness of the specimen was varied from 0.1 to 2 mm, while the width of the specimen was maintained at 1 mm. The size of the stage was 2 × 1 mm (thickness × width) and the temperature was changed from 20 to −60 °C, as shown in Figure 4. It is evident that the stress values of the specimens decrease with increasing distance from the connection between the specimen and stage, as shown in Figure 4a,b. This was result of the thermal expansion rate of the specimen being larger than that of the stage. The smaller deformation value of the stage at low temperature induces the bottom of the specimen under a tensile state, while the tensile stress rapidly declines in the upward direction. Thus, selecting a thicker specimen seems to be able to eliminate the internal stress affecting to the surface of specimen, as shown in Figure 4a,b.

However, with an increase in thickness, the internal stress reappears at the center of the specimen, as shown in Figure 4c,d. The stress state converts from tensile to compressive stress, while the value of the compressive stress is relatively small. This can be easily understood as a limited bending process of the specimen. When the specimen has sufficient stiffness, as well as thickness, the stretching state of the bottom side will inevitably lead to compression state on the surface side of the specimen. At a constant specimen width, the compressive stress at the center of the specimen continues to decrease but it always exists when the thickness increases. It can also be observed from the comparison of each image in Figure 4 that the stress values of the specimens with different thicknesses in the same area are slightly different. This is due to the fact that as the thickness of the specimen increases, the elastically deformed area increases. It reduces the stress value of each area to a certain extent and the specimen is also subjected to stress caused by the expansion in other directions. However, these stresses are small, and do not affect the trend of stress changes in the entire area. From the results obtained from Figure 4, it can be concluded that it is unreasonable to eliminate the internal stress inside the specimen’s surface through controlling the specimen thickness, as the stress-free position is actually an unstable transition state between tensile and compressive stress. A potential solution is selecting the edge region of the specimen’s surface to conduct indention, as the corresponding internal stress is relatively small (especially when the thickness of the specimen is sufficient).

**Figure 5:**
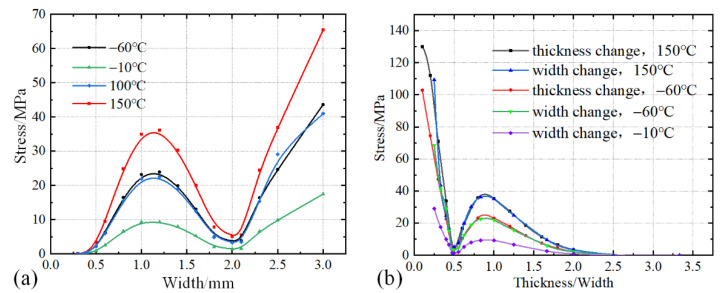
Relationship between internal stress at midpoint and (**a**) width, (**b**) thickness/width value of specimen at different temperatures.

To further explore the influence of the shape of the specimen on the internal stress, the width of the specimen was varied from 0.2 to 3 mm. The variable temperature test was conducted at 20 to −10 °C, 20 to −60 °C, 20 to 100 °C, and 20 to 150 °C. The stress values at the midpoint on the top surface of the specimen were extracted to construct a stress–width curve, as shown in Figure 5a. It is evident that the stress value at indentation point first increases, then decreases to zero, and finally increases in general with the increase of the width. This is not related to the temperature of the environment. The law of stress changing with the width of the specimen is opposite to the law of stress changing with the thickness of the specimen, as shown in Figure 4. This was due to the fact that the increase of width is another form of decrease of length from the proportion perspective. Thus, the Von Mises stress of the midpoint versus the thickness-to-width ratio was calculated, as shown in Figure 5b. It can be observed that at the same temperature, whether it is the data of the thickness or width change tests, the constructed curves overlapped. Thus, the effect of the sample shape on the internal stress is essentially the thickness-to-width ratio. This should be selected to be as large as possible.

**Figure 6:**
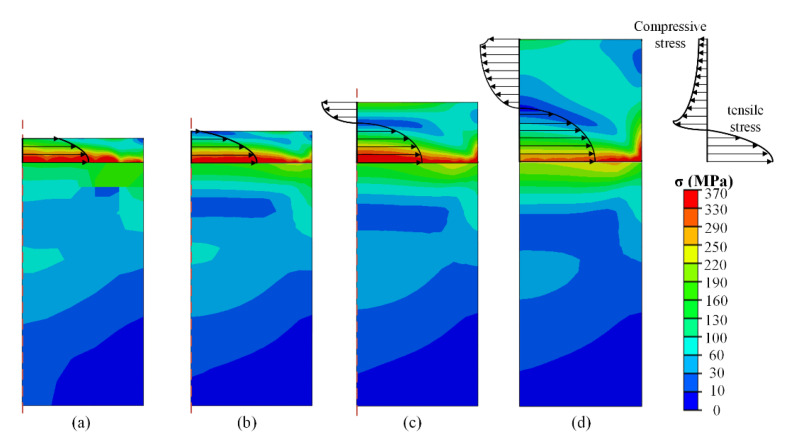
Stress distribution in specimen with thickness of specimen of (**a**) 0.2 mm; (**b**) 0.26 mm; (**c**) 0.5 mm; (**d**) 1 mm at −60 °C at the condition of 720 GPa elastic modulus of specimen.

Besides the shape factor, the influence of the mechanical parameters of the specimen should also be considered. Figure 6 shows the stress distribution of the temperature-change test after the elastic modulus had been magnified ten times to 720 GPa compared with Figure 4, while the shape of specimen and test temperature was the same. It is evident that the stress distributed in Figure 6 is significantly more intense than in Figure 4, indicating that the elastic modulus of the specimen is a significant influential factor for the internal stress distribution. Specimen with higher elastic modulus can transfer stress more effectively inside the material, and the stress around connection surface even closes to the yield stress. However, this is an ideal scenario without glue to connect the specimen and stage. This will be discussed in the next section. From Figure 6, we conclude that the aforementioned method of “selecting edge region to conduct indentation” is unfeasible, as the entire surface can be influenced by internal stress when the specimen has a high elastic modulus.

**Figure 7:**
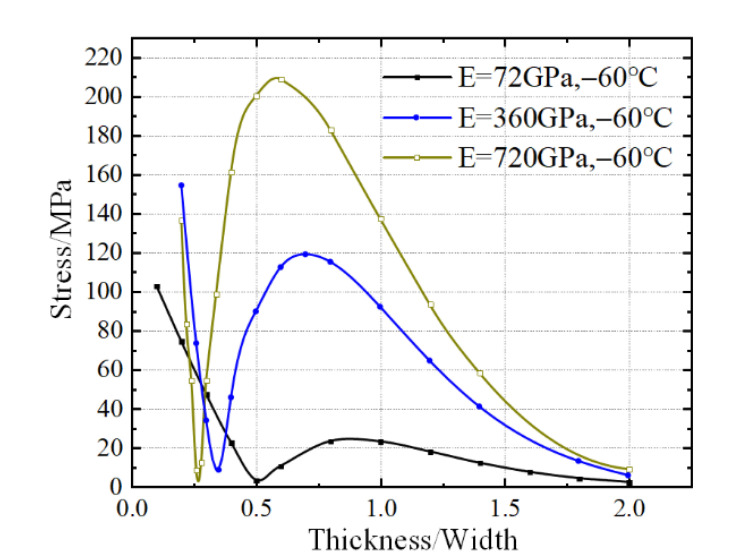
Internal stress at the midpoint of specimen surfaces varying with thickness/width values of specimen at different elastic moduli of the specimen at −60 °C.

Figure 7 shows the internal stress at the midpoint of the surface inside specimens having different elastic moduli with the variation of thickness-to-width ratio of specimen at −60 °C. It is evident that the elastic modulus of specimen not only affects the value of internal stress but also changes the abscissa of the inflection points in the curves. This cannot be achieved by temperature changing as shown in Figure 5b. Thus, it is difficult to select specimens with specific thickness-to-width ratios to avoid the internal stress influence in a thin specimen, as the value at a ratio of 0.5 is nearly the minimum and maximum values at 72 GPa and 720 GPa, respectively. The parameters of the specimen, including the shape and mechanical properties, affect the value and distribution of the internal stress simultaneously. Meanwhile, it is impossible to change the intrinsic characteristic, as well as elastic modulus, to reduce the internal stress. The only viable solution seems to ensure sufficient thickness of the specimen to reduce the stress transmission from the bonding surface. However, the internal stress cannot be completely eliminated due to the bending-like behavior.

### 4.2. External Conditions

#### 4.2.1. Fixed Stage

**Figure 8:**
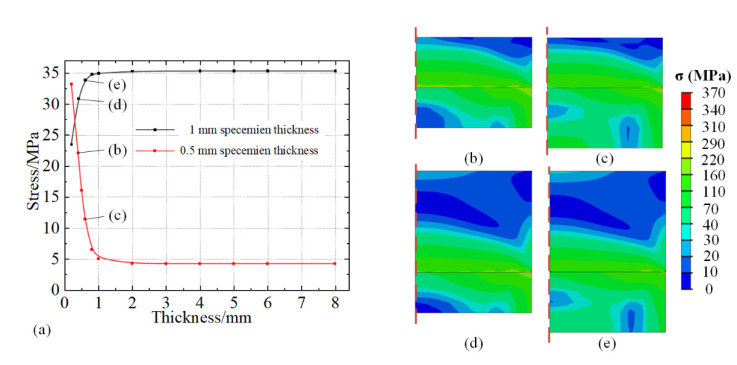
(**a**) Internal stress at the midpoint of specimen with different thicknesses of the stage, and stress distribution with thickness of the stage at (**b**,**d**) 0.4 mm, (**c**,**e**) 0.6 mm, thickness of specimen at (**b**,**c**) 0.5 mm, and (**d**,**e**) 1.0 mm at 150 °C.

To explore the influence of the dimension parameters of the stage on the stress distribution inside the specimen, the thickness of the stage was changed from 0.2 to 8 mm and the tests were conducted at 20 to 150 °C. Meanwhile, two kinds of thickness of specimens, 0.5 and 1 mm, were tested. The corresponding Von Mises stresses are shown in Figure 8a. It is evident that the internal stress at midpoint reaches a steady state with the thickness continues to grow. This indicates that as long as the stage has a certain thickness (more than 1 mm), the influence induced by stage thickness is constant. However, it can also be observed that the thickness of the stage has an opposite effect on the two specimens. When the thickness of the specimen is 0.5 mm, the stress decreases first and then tends to be stable, and the stable value is 4.226 MPa. While the stress increases first and stables at 35.368 MPa in 1 mm specimen. The differences of the stable stresses can be explained by the tensile and compressive stress states induced by the thickness changes of the specimen discussed in Section 4.1. However, the reason for the opposite trend of the stress variation at the initial change of stage thickness is unclear. Four images of stress distribution were extracted from Figure 8a and are shown in Figure 8b–e, which performed a rapidly changing range within 1 mm thickness of the fixed stage. It is evident that the general distribution of internal stress inside the specimen and the stage is similar at the same region of specimen thickness. With the increase of stage thickness, transmission of compressive stress to the surface of the specimen is inhibited to some extent, as shown in Figure 8b,c. This is due to the fact that the elastic strain in the stage caused by mismatched thermal expansion can be carried out in a larger volume with the thickness of stage increasing. The external compressive force applied to the bottom of the specimen slightly decreases for stage thickness within 1 mm. With the thickness continuing to increase, the distribution of the elastic deformation inside the stage transfers sufficiently. This is observed in Figure 4 and Figure 6. The overall deformation tends to be stable. For a scenario with a 1 mm thick specimen, the external stress at the midpoint of surface is under tensile stress as discussed in Section 4.1. This is in the same direction of the influence of increasing stage thickness, inducing a result of increasing internal stress. However, a fixed stage with the thickness within 1 mm is usually not considered, as damage and plastic deformation may occur on the stage during the disassembly process of the specimen. A stage with sufficient thickness is conducive to stabilize the stress distribution inside the specimen.

**Figure 9:**
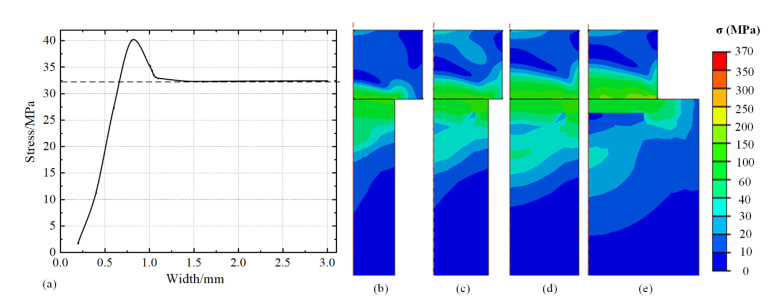
(**a**) Internal stress at midpoint of specimen with different width of stage, and stress distribution with width of stage at (**b**) 0.6 mm, (**c**) 0.8 mm, (**d**) 1 mm, and (**e**) 1.6 mm at 150 °C.

The aforementioned discussion is valid when the widths of the specimen and stage are the same (this is hard to achieve in actual experiments). Thus, the width of the stage is set to change to explore the stress distribution when the width of the specimen and the stage are different. To avoid the influence caused by the thickness of the stage, the thickness of the stage was set to 4 mm, and the size of the specimen was set to 1 × 1 mm. Figure 9 shows the variation of the midpoint stress of the specimen with width of the stage in the range 0.2–3 mm. It is evident that the stress of the specimen increases with the width of stage increasing and reaches maximum value at approximately 0.8 mm of the stage width. Then the stress declines and stabilizes at value of approximately 32.4 MPa. Images of the stress distribution extracted from Figure 9a are shown in Figure 9b–e, with the width of stage at 0.6, 0.8, 1, and 1.6 mm, respectively. It is observed that when the width of the stage is significantly smaller than the specimen, most areas of the specimen can be fully expanded and the tensile stress is reduced, inducing the influence area of compressive stress on the connecting surface is small. Thus, the stress value on the top surface of the specimen is small. As for the width of the stage, it is smaller than but close to the width of the specimen (0.8 mm). The insufficient expansion area becomes larger, resulting in an increase in the superimposed stress. With the width of the stage slightly increasing (0.8–1 mm), the area of compressive stress caused by the connection surface becomes larger. The superimposed stress is offset to a certain extent. The influence area of stress generated at the corners of the stage increases and is concentrated. It formed an angle of more than 90° with the tensile stress, resulting in decreasing superimposed stress. With the width of the stage further increasing (more than 1 mm), most area of the stage is sufficiently expanded and the area affected by the connection surface remains unchanged. This is similar to the results of the thickness of the stage in Figure 7.

#### 4.2.2. Adopted Glue

**Figure 10:**
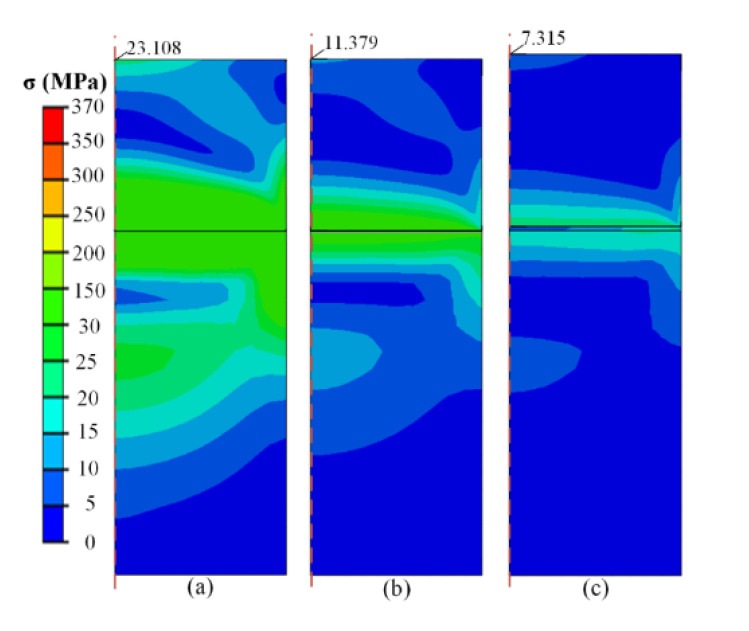
Stress distribution in specimen with thickness of glue of (**a**) 0 nm (**b**) 600 nm, and (**c**) 2500 nm at −60 °C.

In experimental indentation tests, the specimen and stage are usually connected with glue to fix the specimen. The corresponding main materials of commonly used glue, including epoxy, acrylate and phenolic, were adopted to perform the test. The specific properties of each glue are listed in Table 3. To explore the influence of the glue on the stress in the specimen, the thickness of each glue was varied in the range of 250–2500 nm, while the width of the glue was maintained at 1 mm. The size of the specimen and stage was 1 × 1 and 2 × 1 (thickness × width), respectively. Figure 10 shows the stress distribution adopting epoxy as glue with thicknesses of 0, 600, and 2500 nm, respectively. It is observed that the existence of glue reduces the internal stress both inside the specimen and stage effectively. Glue with 600 nm thickness can reduce the internal stress at the midpoint by half (23.108 MPa to 11.379 MPa), while only a third of the original stress is performed in 2500 nm condition. As the elastic modulus of glue is much less than that of the specimen and the stage, the glue performs as a buffer layer between them and carries out coordinated deformation.

**Figure 11:**
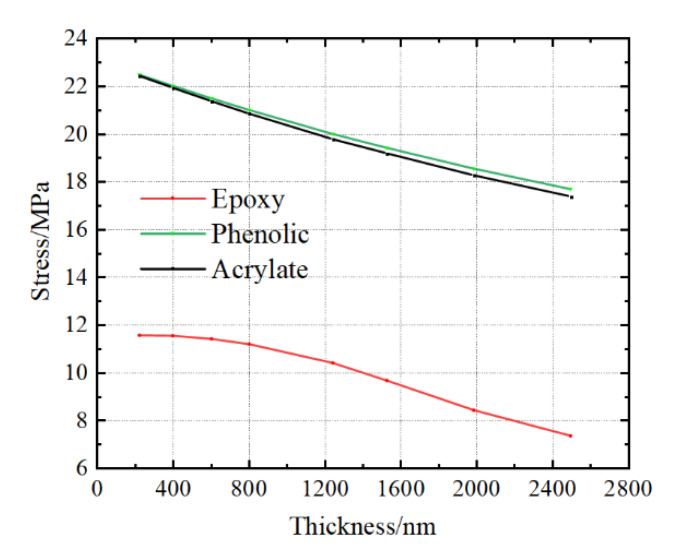
Relationship between the internal stress at the midpoint and thicknesses of different glues at −60 °C.

Figure 11 shows internal stress at the midpoint adopting different thicknesses of glue with epoxy, acrylate and phenolic, respectively. Combining with the data in Table 3, it is evident that the adopted glue with lower elastic modulus has more evident effects of reducing the internal stress both inside the specimen and the stage. The model in Figure 10 is consistent with the real scenario in experimental test. We usually find the debonding phenomenon between the specimen and stage after cyclic temperature tests. Even though we know the effect of the thickness and elastic modulus of adopted glues on the stress distribution, the adhesive ability and heat transfer effect should also be considered.

## 5. Conclusions

In this work, the factors influencing the results of indentation tests at variable temperatures were investigated using the finite element method. The influence of the thermal expansion rate was considered. Relevant factors, such as specimen characteristics, size of stage, and adopted glue, were also explored in detail. The internal stress value is significantly related with the thickness and the ratio of thickness-to-width of the specimen. Both additional tensile and compressive stress could occur inside the indentation surface. Meanwhile, the elastic modulus of specimen determines the absolute value of internal stress. The external conditions, including the size of fixed stage and parameters of glue between specimen and stage, also affect the distribution of internal stress significantly. Thus, the following suggestions for reducing the influence of internal stress are proposed. The most direct and effective method is to select a stage with a coefficient of thermal expansion similar to that of the specimen. A stage made of the same material of the specimen can absolutely eliminate the internal stress. Meanwhile, suitable glue with a smaller Young’s modulus can also effectively reduce the stress inside the specimen. A larger value of the thickness-to-width ratio of specimen and size of fixed stage can inhibit the influence of internal stress to a certain extent.

## Figures and Tables

**Table 1:** Properties of tested materials [20].

Materials	Elastic Modulus (MPa)	Poisson’s Ratio	Yield Stress (MPa)	Thermophysical Properties	
				Temperature (°C)	Thermal Expansion Coefficient (10^−6^/°C)
AlCu_2.5_Mg	72,000	0.33	370	−50	21.8
				20	23.4
				200	24.5
A333–1.6	206,000	0.3	345	20	8.31
				100	10.99
				200	12.31

**Table 2:** Test results of sample.

Temperature (℃)	Hardness (GPa)	Error Percentage of Hardness (%)	Elastic Modulus (GPa)	Error Percentage of Elastic Modulus (%)
150	1.467	−1.08	100.2	−0.694
20	1.483	0	100.9	0
−60	1.517	2.29	102.7	1.78
−196	1.547	4.32	104.2	3.27

**Table 3:** Properties of tested glues [21].

Material	Epoxy	Acrylate	Phenolic
Yield strength (MPa)	27.9	110	210
Elastic modulus (MPa)	1000	6500	6950
Poisson’s ratio	0.38	0.35	0.35
Thermal expansion coefficient (10^−6^/°C)	67.7	90	25
